# Temperature-Dependent Development, Survival, and Population Growth of *Novius penicillioides*: Implications for Biological Control of *Icerya jacobsoni* Under Climate Warming

**DOI:** 10.3390/insects17101067

**Published:** 2026-10-15

**Authors:** Jing Peng, Jianfeng Liang, Chuyang Huang, Shaukat Ali, Xingmin Wang

**Affiliations:** 1 Department of Entomology, College of Plant Protection, South China Agricultural University, Guangzhou 510642, China; pengjing17@scau.edu.cn (J.P.); liangjeff@stu.scau.edu.cn (J.L.); huangcy@stu.scau.edu.cn (C.H.); aliscau@scau.edu.cn (S.A.); 2 Engineering Research Center of Biological Control, Ministry of Education, Guangzhou 510642, China

**Keywords:** *Novius penicillioides*, *Icerya jacobsoni*, thermal adaptation, life table, biological control, development, fecundity

## Abstract

The cottony cushion scale *Icerya jacobsoni* is a serious pest of agriculture and forestry, and the ladybird *Novius penicillioides* is an important predator of this pest. Temperature strongly affects insect development and reproduction, but the thermal biology of *N. penicillioides* remains unknown. Here, *N. penicillioides* was reared at six constant temperatures (17–32 °C) for immature development and survival, and at five temperatures (17–29 °C) for adult reproduction and life table analysis. Developmental duration decreased with rising temperature, whereas survival and fecundity were highest at 20–26 °C. Egg hatch rate fell to 0.25 at 32 °C, and fecundity at 29 °C was 84.3% lower than at 20 °C. The intrinsic rate of increase and finite rate of increase peaked at 26 °C (0.091 and 1.095), identifying this as the optimal temperature for population growth. The lower developmental threshold for the egg-to-adult period was 3.76 °C, with a degree-day requirement of 476.19 degree-days. At 32 °C, only 18% of immatures reached adulthood and the few surviving females failed to reproduce, indicating that the upper thermal limit for population growth lies between 29 and 32 °C. Given that natural enemies often have lower optimal temperatures than their prey, a thermal asymmetry may exist between *N. penicillioides* and *I. jacobsoni*, and continued warming could reduce its summer control efficacy. These findings provide key thermal parameters for optimizing mass rearing and field release strategies of this predator.

## Simple Summary

Scale insects are major pests of agriculture, forestry, and ornamental plants worldwide, and the ladybird *Novius penicillioides* is an important natural enemy of the cottony cushion scale *Icerya jacobsoni*. Because insects are cold-blooded, their growth, survival, and reproduction depend heavily on temperature, yet the thermal limits of this ladybird have not been studied. We reared *N. penicillioides* at six constant temperatures from 17 to 32 °C and recorded how long it took to develop and how many survived. At five of these temperatures (17–29 °C), we also recorded how many eggs each female laid and how quickly its population grew. The ladybird developed fastest at the highest temperatures, but it survived and reproduced best between 20 and 26 °C. At 29 °C, egg production dropped sharply, and at 32 °C, egg hatching fell dramatically, indicating that these temperatures are close to the upper limit this insect can tolerate. Our results suggest that this ladybird may cope less well with hot weather than its prey, which means it could become less effective at controlling scale insects as the climate warms. We recommend releasing it when average daily temperatures are between 20 and 26 °C, and avoiding releases during hot summer periods.

## 1. Introduction

Scale insects (Coccoidea) constitute a major group of pests affecting agriculture, forestry, and horticultural production worldwide [1]. In recent years, species of the genus Icerya have emerged as serious threats to a wide range of crops and ornamental plants, with particularly severe impacts in forestry [2,3]. *Icerya jacobsoni* Green (Hemiptera: Monophlebidae) is a highly polyphagous pest that causes substantial damage to economically important crops and landscape plants across its distribution range. Both nymphs and adult females aggregate on tender shoots and the undersides of leaves, where they feed on plant sap. Their honeydew secretions promote the development of sooty mold, which impairs photosynthesis and ultimately leads to leaf yellowing, premature abscission, and in severe cases, plant death [4]. In China, *I. jacobsoni* has become established in several provinces, including Guangdong, Guangxi, and Fujian, with recorded hosts spanning more than 20 plant families, such as Magnoliaceae, Moraceae, Euphorbiaceae, and Myrtaceae [5,6]. This broad host range and expanding distribution pose a persistent threat to agricultural and forestry production.

Chemical insecticides remain the primary tactic for managing scale insect pests. However, the cryptic habits, hydrophobic waxy secretions, and symbiotic gut microorganisms of these insects substantially reduce the efficacy of conventional chemical control [7,8]. The waxy layer covering the insect body limits the penetration of insecticide solutions, which often compels growers to increase application rates and, consequently, accelerates the development of resistance [8]. Moreover, *I. jacobsoni* is covered with waxy secretions at all developmental stages and closely resembles the related species *I. aegyptiaca* in morphology, which frequently leads to misidentification and further complicates monitoring and control efforts [6]. Overreliance on chemical pesticides also poses risks to human and animal health, contributes to environmental bioaccumulation, and harms natural enemy populations [8]. Consequently, biological control using natural enemies has become an increasingly important component of sustainable management strategies for *I. jacobsoni*.

Ladybirds are important predatory natural enemies of scale insects and have a long history of application in biological control. The successful introduction of *Novius cardinalis* for the control of the cottony cushion scale, *Icerya purchasi*, represents a classic example of biological control that has remained effective in many regions worldwide since its first application in 1888 [9]. *Novius penicillioides* Tao & Wang (Coleoptera: Coccinellidae) is an important predator of *I. jacobsoni* [10]. Larvae of this ladybird preferentially attack nymphs of *I. jacobsoni*, with a marked preference for second-instar nymphs, often leaving only the empty integument after feeding [3]. Adults also consume eggs and nymphs, frequently causing body fluid outflow and coagulation during feeding. A single larva of *N. penicillioides* can consume 25 to 40 eggs or nymphs of *I. jacobsoni* per day, indicating considerable predatory potential. Elucidating the biological characteristics of *N. penicillioides* and its responses to environmental factors is therefore essential for optimizing its use in biological control programs.

Insects are ectotherms, and their body temperature and life activity rhythms are closely dependent on ambient temperature [11]. Temperature regulates not only developmental rate, survival, fecundity, and population growth parameters but also geographic distribution and seasonal occurrence patterns [12]. Ladybird beetles exhibit considerable plasticity in reproductive output and development in response to thermal variation, with constant and fluctuating temperatures differentially affecting their fitness traits [13]. Moreover, thermal conditions during larval development modulate developmental plasticity, including the rate of limb regeneration [14]. The four developmental stages—egg, larva, pupa, and adult—differ in their thermal sensitivity, with eggs being the most sensitive and adults the most tolerant to cold stress [15], making ladybirds suitable subjects for investigating insect thermal adaptation. Under global climate warming, the increasing frequency of extreme heat events may profoundly affect the pest control efficacy of predatory ladybirds. Therefore, a systematic assessment of how temperature influences the ecological adaptation of predatory ladybirds is an important scientific foundation for optimizing biological control strategies under climate change.

Previous studies have demonstrated that the inhibitory effects of high-temperature stress on insect reproduction are widespread. When ambient temperature exceeds the optimal range, insects tend to allocate more energy to thermotolerance, such as the synthesis of heat shock proteins, thereby diverting resources away from reproduction and creating a survival-reproduction trade-off [16]. High temperatures can also directly impair oocyte development and inhibit vitellogenin synthesis, leading to reduced fecundity and lower egg hatch rates [17]. The effects of temperature on the biological traits of coccinellids have been documented in several species. For example, pupal heat stress significantly reduces cumulative fecundity, egg hatch rate, and adult longevity in *Harmonia axyridis* [18]. In *Coccinella septempunctata*, extreme high temperatures significantly decrease adult and egg survival, fecundity, and egg hatch rate, with effects intensifying as the duration of exposure increases [19]. However, for *N. penicillioides*, a dominant natural enemy of *I. jacobsoni*, no systematic study has examined its development, survival, reproduction, and population growth parameters across a temperature gradient. In particular, its adaptive responses across a broad thermal range of 17–32 °C remain unexplored. Notably, the optimal developmental temperature of natural enemies is often lower than that of their host pests, making them more susceptible to suppression under high-temperature conditions [20]. Thus, under climate warming, the control efficacy of ladybirds and other natural enemies may face greater challenges, underscoring the urgent need for systematic thermal adaptation studies to assess this risk.

In the present study, six constant temperature treatments (17, 20, 23, 26, 29, and 32 °C) were established to systematically measure the developmental duration, survival rate, adult fecundity, and longevity of *N. penicillioides*. An age-specific life table was constructed to calculate the net reproductive rate, intrinsic rate of increase, finite rate of increase, and mean generation time. This study aimed to: (1) clarify the comprehensive effects of temperature on the development, survival, and reproduction of this ladybird; and (2) identify the optimal temperature range for population growth and the upper thermal tolerance threshold. The results will provide a scientific basis for optimizing mass-rearing protocols and field release strategies of *N. penicillioides* based on climatic data.

## 2. Materials and Methods

### 2.1. Insect Rearing

The stock colony of *I. jacobsoni* used in this study was obtained from the Engineering Research Center of Biological Control at South China Agricultural University, where it had been maintained on *Codiaeum variegatum* (L.) for multiple generations. The predator *N. penicillioides* was sourced from the same institute’s greenhouse colony and was reared on *I. jacobsoni* infesting *C. variegatum*. Prior to the experiments, both *I. jacobsoni* and *N. penicillioides* had been continuously reared on *C. variegatum* for several generations to ensure acclimatization to the experimental conditions. All insect cultures were maintained in an environmental chamber set at 26 ± 1 °C, 75 ± 10% relative humidity, and a photoperiod of 14 L: 10 D. The *C. variegatum* plants used for rearing were cultivated in plastic pots with a diameter of 15 cm and were fertilized as needed with a compound fertilizer containing N:P:K at a ratio of 13:7:15 to sustain healthy plant growth.

### 2.2. Development and Survival of N. penicillioides Immatures Under Different Temperatures

To evaluate the effect of temperature on the developmental duration and survival of *N. penicillioides*, eggs laid on the same day were placed on moistened filter paper in Petri dishes. Six constant temperature treatments (17, 20, 23, 26, 29, and 32 °C) were set in separate incubators, with 100 eggs observed per temperature. Hatching was checked daily. Upon emergence, neonates were individually transferred to fresh leaf disks bearing nymphs of *I. jacobsoni* and reared singly. Except during the pupal stage, leaf disks were replaced daily. The developmental progress and survival of each immature stage were monitored daily until adult emergence. Each temperature treatment was replicated three times.

### 2.3. Reproduction and Adult Longevity of N. penicillioides Under Different Temperatures

Five constant temperature treatments (17, 20, 23, 26, and 29 °C) were established to assess the reproductive performance and adult longevity of *N. penicillioides*. The 32 °C treatment was excluded from the adult experiment because adult emergence at this temperature was extremely low, making it impossible to obtain sufficient mating pairs for replication. Newly emerged adults were paired and reared individually in Petri dishes. Fresh prey and leaf disks were supplied daily, and the number of eggs laid and adult survival were recorded each day until the death of all individuals.

### 2.4. Data Analysis

Developmental duration, adult longevity, preoviposition period, and total fecundity per female of *N. penicillioides* were compared among temperature treatments using one-way analysis of variance (ANOVA), followed by Duncan’s multiple range test for mean separation when significant differences were detected (*p* < 0.05). Survival rates, which are proportional (binomial) data, were analyzed using a generalized linear model (GENLIN) with a binomial distribution and a logit link function. Pairwise comparisons among temperature treatments were performed using the sequential Bonferroni method, with differences considered significant at *p* < 0.05. The relationship between developmental rate and temperature for each life stage and the entire generation was described by a linear regression model: V(t) = (1/K) × T − C/K, where V(t) denotes the developmental rate in days at temperature T (°C), K represents the required heat units in degree-days, and C is lower developmental threshold [21]. For stages where the linear model was not applicable, a Logistic model was used: V(T) = k/(1 + e^a−bT^), where k is the maximum developmental rate and a and b are model coefficients.

Life table parameters were calculated according to the procedures of Birch [22]. The following equations were employed:

*R*_0_ = ∑*l_x_m_x_*, (1)

*T* = ∑*xl_x_m_x_*/*R*_0_, (2)

*r_m_* = *lnR*_0_/*T*, (3)

*λ* = *exp* (*r_m_*), (4)

where *x* denotes the age of *N. penicillioides* measured in days, $l_{x}$ is the survival at a specific time *x* and $m_{x}$ is the average number of female offspring generated by each surviving female adult within the age interval *x*. *T* refers to the mean generation time, defined as the average age at which females produce offspring. $R_{0}$ is the net reproductive rate, representing the average number of offspring that an individual in the population is expected to produce over its lifetime. The intrinsic rate of increase ($r_{m}$) describes the rate at which a population expands in size in the absence of density-dependent factors that might otherwise regulate it, while *λ* represents the finite rate of increase, indicating the factor by which the population size must be multiplied to project the population size in the subsequent time unit, assuming a stable age distribution.

## 3. Results

### 3.1. Effects of Temperature on Development and Survival of N. penicillioides

Temperature exerted a significant influence on the developmental duration of each immature stage as well as the overall preimaginal period of *N. penicillioides* (Table 1). Across the tested temperature range, developmental times generally decreased with increasing temperature; however, the magnitude of this reduction varied among different thermal intervals. The egg stage lasted longest at 17 °C (11.22 ± 0.24 d) and progressively shortened as temperature rose, reaching a minimum of 4.16 ± 0.30 d at 32 °C, with no significant difference observed between 29 °C and 32 °C. The combined larval duration (four instars) was greatest at 17 °C (19.70 ± 0.34 d) and declined markedly with increasing temperature, reaching 9.99 ± 0.14 d at 23 °C; at 26 °C, it increased to 12.24 ± 0.42 d, and at 29 °C and 32 °C, the durations were 8.24 ± 0.43 d and 10.27 ± 0.55 d, respectively. The pupal stage shortened considerably between 17 °C and 23 °C (from 7.58 ± 0.13 d to 4.70 ± 0.19 d), whereas only minor changes were recorded between 26 °C and 32 °C (ranging from 5.96 ± 0.14 d to 4.07 ± 0.33 d). The total developmental period from egg to adult was longest at 17 °C (38.50 ± 0.36 d) and shortest at 23 °C (21.86 ± 0.15 d), slightly extended to 23.75 ± 0.44 d at 26 °C, and further reduced to 18.30 ± 0.46 d and 18.51 ± 0.51 d at 29 °C and 32 °C, respectively, with no significant difference between the two highest temperatures. Notably, the fourth-instar duration did not follow the overall decreasing trend with temperature. It remained relatively short at 17–23 °C (4.72 ± 0.17, 3.14 ± 0.08, and 3.14 ± 0.11 d, respectively), increased markedly to 6.69 ± 0.26 d at 26 °C, and then decreased again to 3.09 ± 0.21 d and 4.14 ± 0.22 d at 29 °C and 32 °C, respectively.

Temperature significantly affected egg hatch rate and total preimaginal survival from egg to adult, whereas its effect on larval survival at individual instars and pupal survival was not significant (Table 2). Egg hatch rate was highest at 20 °C (0.99 ± 0.01), followed by 23 °C (0.96 ± 0.02) and 26 °C (0.95 ± 0.02), and declined to 0.89 ± 0.03 at 17 °C and 0.81 ± 0.04 at 29 °C, reaching a minimum of 0.25 ± 0.04 at 32 °C (Wald χ^2^ = 146.307, df = 5, *p* < 0.001). In contrast, survival of first-, second-, third-, and fourth-instar larvae and pupae remained consistently high across all temperatures (mostly > 0.90), with no significant differences detected among temperature treatments (*p* > 0.05). Total survival from egg to adult was highest at 23 °C and 26 °C (both 0.86 ± 0.04), followed by 20 °C (0.81 ± 0.04) and 17 °C (0.75 ± 0.04), and declined sharply at 29 °C (0.62 ± 0.05) and 32 °C (0.18 ± 0.04) (Wald χ^2^ = 114.440, df = 5, *p* < 0.001).

### 3.2. Degree-Day Requirement of N. penicillioides

The fitted relationships between developmental rate and temperature for each life stage are presented in Table 3. For the egg, first-, second-, and third-instar larvae, and the overall egg-to-adult period, developmental rate increased linearly with temperature, with R^2^ values ranging from 0.8752 to 0.9790. Based on the linear model, the lower developmental thresholds for the egg, first-, second-, and third-instar larvae, and the egg-to-adult period were estimated as 5.73, 10.41, 9.01, 11.09, and 3.76 °C, respectively, with corresponding degree-day requirements of 119.05, 31.35, 27.93, 32.47, and 476.19 degree-days.

For the fourth-instar larva and pupa, however, developmental rate did not show a significant linear relationship with temperature, with R^2^ values of only 0.0119 and 0.5925, respectively. The fourth-instar duration at 26 °C (6.69 d) was abnormally extended, causing the developmental rate at this temperature to deviate markedly from the linear trend. The pupal duration at 26 °C (5.96 d) was longer than at 23 °C (4.70 d) and 29 °C (4.73 d), exhibiting a non-monotonic fluctuation that likewise departed from linearity. Consequently, the lower developmental threshold and degree-day requirement could not be reliably estimated for these two stages using the linear model. The Logistic model provided a substantially better fit for the fourth instar and pupa, with R^2^ values of 0.9295 and 0.9850, respectively. Therefore, the relationship between developmental rate and temperature for these two stages is described using the Logistic model, and their linear-model estimates of lower developmental threshold and degree-day requirement are not reported.

### 3.3. Effects of Temperature on Adult Longevity and Reproduction of N. penicillioides

Temperature significantly influenced the preoviposition period, fecundity, and adult longevity of *N. penicillioides* (Table 4). The preoviposition period was shortest at 20 °C (6.5 ± 0.43 d) and remained relatively short at 23 °C (6.8 ± 0.39 d) and 26 °C (7.6 ± 0.40 d), with no significant differences among these three treatments. It was slightly longer at 17 °C (7.90 ± 0.34 d) and increased markedly to 12.9 ± 0.64 d at 29 °C, which was significantly longer than all other treatments. Total fecundity per female was highest at 20 °C (343.30 ± 35.68 eggs), significantly surpassing all other treatments. At 17 °C, 23 °C, and 26 °C, fecundity values reached 247.80 ± 29.10, 209.00 ± 30.78, and 252.40 ± 25.24 eggs, respectively, with no significant differences among these three temperatures. At 29 °C, however, fecundity dropped drastically to 53.90 ± 15.93 eggs. The relationship between temperature (T) and total fecundity per female (Y) was described by a quadratic equation: Y = −1162.7 + 141.87T − 3.413T^2^ (r = 0.9381, *p* < 0.05). Adult longevity declined with rising temperature: it was longest at 20 °C (128.40 ± 9.16 d), followed by 17 °C (112.30 ± 6.62 d), with no significant difference between these two treatments; it decreased significantly to 91.10 ± 7.01 d at 23 °C and further to 85.00 ± 8.84 d and 72.00 ± 7.77 d at 26 °C and 29 °C, respectively, with the latter two not significantly different from each other.

**Figure 1:**
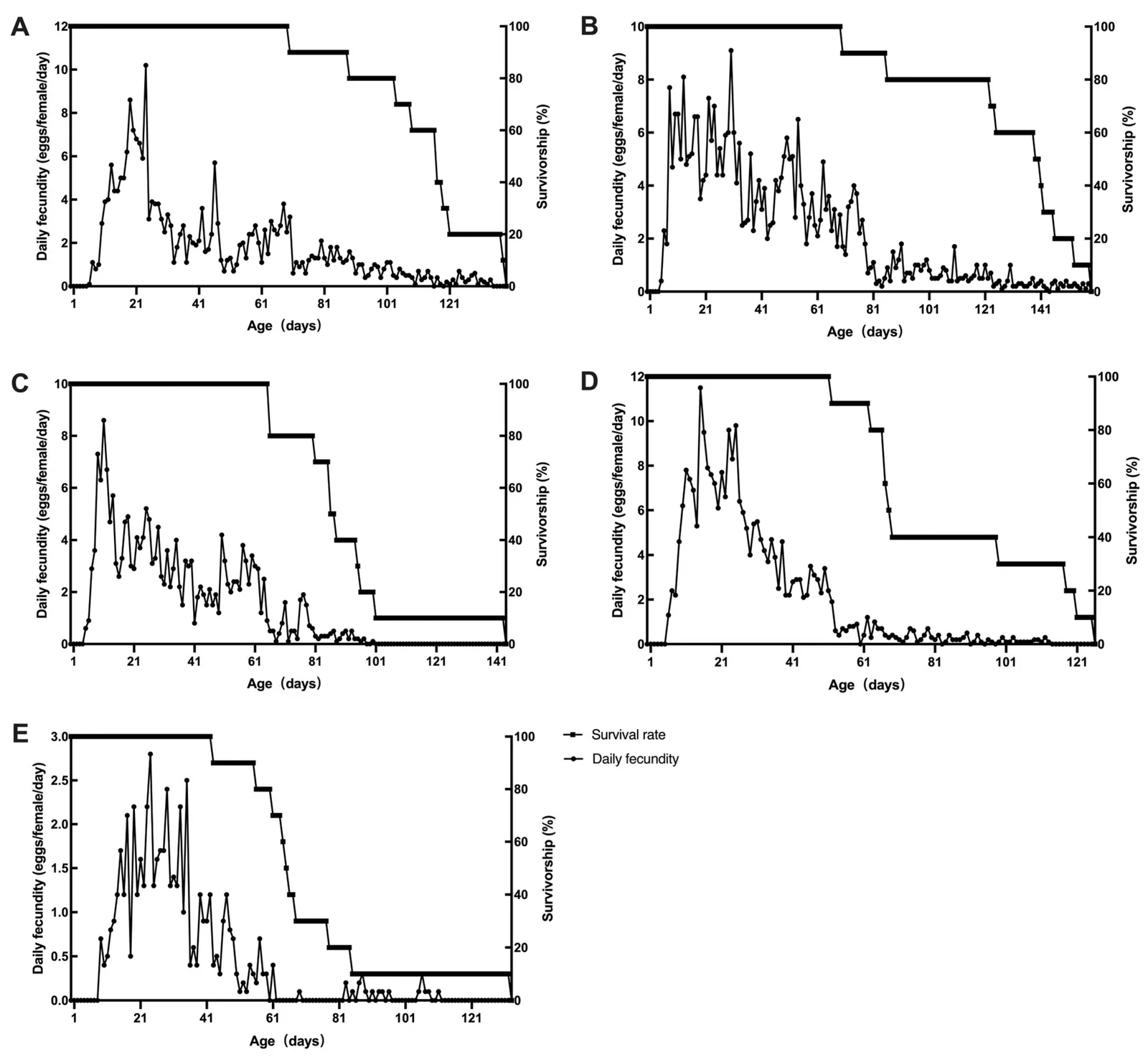
Daily fecundity and survival rate of adult females of *N. penicillioides* at different temperatures. (**A**): 17 °C; (**B**): 20 °C; (**C**): 23 °C; (**D**): 26 °C; (**E**): 29 °C.

Daily oviposition patterns differed markedly among temperature treatments (Figure 1). At 20 °C, egg-laying extended over the longest period, with relatively high daily fecundity maintained during the early and middle stages of adult life. At 26 °C, oviposition was concentrated in the early and middle stages, with a pronounced peak during the early adult stage. At 23 °C, daily fecundity peaked early and remained elevated during the early adult stage. At 17 °C, oviposition was more evenly distributed, with several moderate peaks. At 29 °C, daily fecundity remained low throughout the reproductive period and fluctuated considerably. Adult mortality was not concentrated during the preoviposition period at any temperature. At 17 °C and 20 °C, survival remained high until approximately 80–100 days of age before declining, whereas at 23 °C, 26 °C, and 29 °C, mortality became more pronounced between 70 and 80 days of age, corresponding to the mid-oviposition period.

### 3.4. Life Table Parameters of N. penicillioides at Different Temperatures

Life table parameters of *N. penicillioides*, including the net reproductive rate (R_0_), intrinsic rate of increase (r_m_), finite rate of increase (λ), and mean generation time (T), were calculated for the five temperatures at which reproduction occurred (17, 20, 23, 26, and 29 °C) based on the age-specific life table theory (Table 5). The net reproductive rate varied substantially across temperatures, reaching its maximum at 20 °C (132.45), followed by 26 °C (105.48), with values of 87.30 and 86.75 at 17 °C and 23 °C, respectively, and dropping to the minimum of 13.68 at 29 °C. The intrinsic rate of increase increased gradually with temperature from 17 °C to 26 °C, peaking at 0.091 at 26 °C, and then decreased to 0.056 at 29 °C, which was identical to the value recorded at 17 °C. The finite rate of increase followed the same trend as the intrinsic rate, being highest at 26 °C (1.095) and equal at 17 °C and 29 °C (1.058). The mean generation time consistently shortened with increasing temperature, from a maximum of 79.72 d at 17 °C to a minimum of 46.32 d at 29 °C.

Considering developmental duration, survival rate, fecundity, and life table parameters collectively, the highest population growth potential, as indicated by the peak intrinsic rate of increase (0.091), was observed at 26 °C, where preimaginal survival (0.86) also remained at relatively high levels. These findings suggest that 26 °C represents the optimal temperature for population increase of this ladybird species. In contrast, the sharp decline in egg hatch rate (0.25) and extremely low overall preimaginal survival (0.18) at 32 °C, together with the severe reduction in fecundity (53.9 eggs per female) at 29 °C, indicate that temperatures in the range of 29–32 °C approach the upper sublethal thermal limits for this species.

## 4. Discussion

In the present study, the developmental duration of *N. penicillioides* generally decreased as temperature increased, which is consistent with the general rule that metabolic rates accelerate with rising temperature within the tolerance range of insects [12,23,24]. An unexpected pattern was observed in the fourth-instar duration, which peaked at 26 °C (6.69 d) and was significantly longer than at both lower (17–23 °C) and higher (29–32 °C) temperatures. We cannot fully resolve the underlying mechanism with the current dataset, and this point warrants further investigation using detailed observations of prepupal behavior and energy reserves. Importantly, this anomaly does not affect the overall conclusion that 20–26 °C is the optimal thermal window for *N. penicillioides*, since the total egg-to-adult duration at 26 °C remained short (23.75 d) and the intrinsic rate of increase was highest at this temperature. Consistent with this overall pattern, both survival and fecundity peaked in the moderate temperature range (20–26 °C), while high temperatures (29–32 °C) caused a sharp decline in egg hatch rate and a substantial reduction in fecundity. This pattern of “accelerated development accompanied by reduced fitness” aligns closely with classical theory on insect thermal adaptation and reveals a trade-off between developmental rate and survival/reproduction. The range of 20–26 °C appears to be optimal for the growth and development of *N. penicillioides*. This result differs from the optimal range of 23–29 °C reported by Yao et al. [25] for *Serangium japonicum*, suggesting that different ladybird species have evolved differentiated thermal adaptation strategies over long-term evolution.

The results of this study are consistent with reports on other coccinellid species. In *Nephus arcuatus*, both the intrinsic rate of increase and the net reproductive rate reached their highest values at 30 °C, but fecundity dropped sharply to 136.6 eggs at 35 °C, indicating a pronounced inhibitory effect of high temperature on reproduction [26]. In *Coccinella septempunctata*, survival and fecundity declined significantly under extreme high temperatures of 36–39 °C [19]. In *Harmonia axyridis*, exposure of pupae to 35 °C resulted in significantly lower cumulative fecundity and egg hatch rate in adults compared with the control group [18]. In the present study, fecundity per female at 29 °C (53.9 eggs) was 84.3% lower than that at 20 °C (343.3 eggs), and the egg hatch rate at 32 °C (0.25) was 73.7% lower than that at 26 °C (0.95). This high-temperature sensitivity is consistent with the above species, indicating that coccinellids exhibit a high degree of conservation in their temperature response patterns. The lower developmental threshold and effective accumulated temperature are useful indicators of the potential distribution of insects [27]. *N. penicillioides* requires a threshold temperature of 3.76 °C and 476.19 degree-days to complete the egg-to-adult period, which differs markedly from the values reported by Yao et al. [25] for *S. japonicum* (lower developmental threshold of 9.41 °C and 285.71 degree-days). This indicates significant variation in thermal requirements among ladybird species. These parameters are reported here for the first time and provide a quantitative basis for predicting the occurrence periods of this species in different climatic regions. It should be noted that the linear degree-day model was not applicable to the fourth instar and pupa, as their developmental rates departed from linearity (R^2^ = 0.0119 and 0.5925, respectively). For these two stages, the Logistic model provided a better fit (R^2^ = 0.9295 and 0.9850). This limitation should be considered when using the thermal constants reported here for phenological prediction.

The sharp decline in fecundity at high temperatures can be explained from the perspective of energy allocation. When ambient temperature exceeds the optimal range, insects tend to allocate more energy to thermotolerance, such as the synthesis of heat shock proteins, thereby diverting resources away from reproductive investment and forming a survival-reproduction trade-off. Transcriptomic studies on *Spodoptera litura* have confirmed that heat shock protein genes are significantly upregulated under thermal stress, whereas reproduction-related genes and cell cycle-related genes are suppressed, indicating that insects undergo metabolic reprogramming from reproductive function toward survival adaptation under heat stress [28]. In addition, high temperatures may directly impair oocyte development or inhibit vitellogenin synthesis, leading to reduced fecundity and lower egg hatch rates [29]. High temperatures can also induce a significant increase in reactive oxygen species (ROS) levels in insects. When the rate of ROS generation exceeds the scavenging capacity of antioxidant enzymes, lipid peroxidation and cell membrane damage occur, which in turn affect the normal function of reproductive tissues [30,31]. In the present study, the inhibitory effect of high temperature on reproduction was manifested in both the extension of the preoviposition period and the shortening of the reproductive duration of females.

The intrinsic rate of increase (r_m_) is the best integrated indicator for evaluating the cumulative effects of temperature on insect populations. In this study, both r_m_ (0.091) and λ (1.095) reached their highest values at 26 °C. Although total fecundity per female was highest at 20 °C (343.3 eggs), the longer mean generation time (70.98 d) limited further increases in r_m_. This indicates that the optimal temperature for reproduction is not equivalent to the optimal temperature for population growth [12,32]. A similar conclusion was reached by Papanikolaou et al. [33] for *Propylea quatuordecimpunctata*, in which the net reproductive rate was highest at 25 °C but the intrinsic rate of increase was highest at 30 °C. Zarghami et al. [26] also reported for *N. arcuatus* that r_m_ was highest at 30 °C, but although development was fastest at 35 °C, fecundity declined substantially. The r_m_ value of *N. penicillioides* at 26 °C (0.091) in this study is similar to those reported by Ren et al. [34] for *Nephaspis oculatus* and by Huang et al. [35] for Axinoscymnus cardilobus at the same temperature, but lower than those reported by Tanigoshi and McMurtry [36] for Stethorus picipes and by Yao et al. [25] for *S. japonicum*. These differences may be related to factors such as prey species, host plants, and predator sex ratio.

As an integrated parameter reflecting developmental rate, survival, and fecundity, r_m_ is the optimal indicator for evaluating the thermal adaptation of natural enemy insects. The results of this study provide key temperature parameters for the mass rearing and field release strategies of *N. penicillioides*: it is recommended to release this ladybird during seasons when the daily mean temperature is 20–26 °C to maximize population establishment and pest control efficacy. When the daily mean temperature in the field consistently exceeds 29 °C in summer, the release of eggs or newly hatched larvae should be avoided, as their survival and subsequent fecundity are extremely low. The successful establishment of any insect population depends on temperature [37], and the temperature parameters obtained in this study can provide a direct basis for the introduction and release of *N. penicillioides*. Notably, the optimal developmental temperature of natural enemies is often lower than that of their herbivorous pests [20]. In this study, the optimal temperature for population growth of *N. penicillioides* was 26 °C, whereas its prey *I. jacobsoni*, a scale insect of tropical origin, may have a higher upper thermal tolerance threshold. This asymmetry in temperature responses between natural enemy and pest implies that under continued climate warming, the control efficacy of *N. penicillioides* against *I. jacobsoni* during hot summer months may be further weakened. Future climate warming may lead to reduced control efficacy of *N. penicillioides* in some low-latitude regions, necessitating the development of adaptive management strategies in advance.

It should be noted that this study was conducted under laboratory conditions with constant temperature, constant humidity, and abundant food, and did not simulate realistic ecological factors such as diurnal temperature variation, ultraviolet radiation, and fluctuations in prey density in the field. Studies on other insects have shown that temperature fluctuations during development can reduce male fitness and limit adaptive potential [38], while daily temperature amplitude significantly affects longevity, fecundity, and thermal tolerance [39]. Mean temperature and temperature variation interact to shape life history strategies, heat tolerance, and body size [40], and temperature effects on fitness extend across developmental stages to adult body mass, longevity, and energy reserves [41]. Constant-temperature experiments tend to overestimate the actual adaptive capacity of insects under high temperatures, because the absence of a nocturnal low-temperature recovery period prevents insects from mitigating daytime heat stress through behavioral regulation, such as migrating to cooler microhabitats [12]. Therefore, the 29–32 °C tolerance threshold obtained in this study should be regarded as an upper reference value for field applications. Future studies should combine field cage experiments with diurnal temperature variation treatments to further verify the field applicability of the temperature thresholds obtained here. Notably, rapid evolution of heat tolerance has been documented in natural insect populations over decadal timescales [42], suggesting that the thermal limits observed in this study may not be fixed and could shift under sustained climate warming.

## 5. Conclusions

This study characterized the thermal adaptation of *N. penicillioides* across the range of 17–32 °C. Developmental duration of all immature stages generally decreased with increasing temperature, whereas the optimal temperatures for survival and reproduction did not coincide. The range of 20–26 °C represented the suitable thermal window, with the highest intrinsic rate of increase (0.091) and finite rate of increase (1.095) recorded at 26 °C, identifying this temperature as optimal for population growth. The lower developmental threshold for the egg-to-adult period was 3.76 °C, with a corresponding degree-day requirement of 476.19 degree-days. Given that the optimal temperature of natural enemies is often lower than that of their prey, a thermal asymmetry may exist between *N. penicillioides* and *I. jacobsoni*, and continued climate warming could compromise the control efficacy of this predator during summer months. Field releases are therefore recommended during seasons when the daily mean temperature ranges from 20 to 26 °C, whereas the release of eggs or newly hatched larvae should be avoided when daily mean temperatures consistently exceed 29 °C. These findings reveal a potential asymmetry in thermal adaptation between the predator and its prey, providing a basis for assessing the stability of biological control under climate warming, as well as key thermal parameters for the mass rearing and release strategy optimization of this natural enemy.

## Figures and Tables

**Table 1:** Developmental time (in days) of different stages of *N. penicillioides* at six constant temperatures (Means ± SE).

Temp.	Egg	Larval Instars				Larva	Pupa	Egg-Adult
		1st Instar	2nd Instar	3rd Instar	4th Instar			
17	11.22 ± 0.24 a	4.87 ± 0.20 a	3.70 ± 0.13 a	6.41 ± 0.11 a	4.72 ± 0.17 b	19.70 ± 0.34 a	7.58 ± 0.13 a	38.50 ± 0.36 a
20	7.91 ± 0.25 b	3.33 ± 0.08 b	2.59 ± 0.16 b	3.59 ± 0.09 b	3.14 ± 0.08 c	12.64 ± 0.18 b	7.34 ± 0.31 a	27.89 ± 0.55 b
23	7.17 ± 0.16 c	2.48 ± 0.06 c	1.82 ± 0.11 c	2.56 ± 0.12 c	3.14 ± 0.11 c	9.99 ± 0.14 c	4.70 ± 0.19 c	21.86 ± 0.15 d
26	5.55 ± 0.14 d	1.88 ± 0.10 d	1.67 ± 0.07 c	2.01 ± 0.11 d	6.69 ± 0.26 a	12.24 ± 0.42 b	5.96 ± 0.14 b	23.75 ± 0.44 c
29	5.32 ± 0.04 d	1.76 ± 0.11 d	1.43 ± 0.08 c	1.97 ± 0.09 d	3.09 ± 0.21 c	8.24 ± 0.43 d	4.73 ± 0.15 c	18.30 ± 0.46 e
32	4.16 ± 0.30 e	1.64 ± 0.10 d	1.75 ± 0.17 c	2.75 ± 0.32 c	4.14 ± 0.22 c	10.27 ± 0.55 c	4.07 ± 0.33 c	18.51 ± 0.51 e
*F*; *p*	148.72; <0.001	108.35; <0.001	46.364; <0.001	106.67; <0.001	59.203; <0.001	115.43; <0.001	42.796; <0.001	311.02; <0.001

Means in the same column followed by the same letters are not significantly different (*p* < 0.05).

**Table 2:** Survival of different stages of *N. penicillioides* at six constant temperatures (Means ± SE).

Temp.	Egg	Larval Instars				Pupa	Egg-Adult
		1st Instar	2nd Instar	3rd Instar	4th Instar		
17	0.89 ± 0.03 b	0.90 ± 0.03 a	1.00 ± 0.00 a	0.95 ± 0.02 a	1.00 ± 0.00 a	0.99 ± 0.01 a	0.75 ± 0.04 ab
20	0.99 ± 0.01 a	0.89 ± 0.03 a	0.99 ± 0.01 a	0.96 ± 0.02 a	0.99 ± 0.01 a	0.98 ± 0.02 a	0.81 ± 0.04 a
23	0.96 ± 0.02 ab	0.92 ± 0.03 a	0.98 ± 0.01 a	1.00 ± 0.00 a	1.00 ± 0.00 a	1.00 ± 0.00 a	0.86 ± 0.04 a
26	0.95 ± 0.02 ab	0.96 ± 0.02 a	1.00 ± 0.00 a	0.99 ± 0.01 a	0.98 ± 0.02 a	0.98 ± 0.01 a	0.86 ± 0.04 a
29	0.81 ±0.04 c	0.89 ± 0.04 a	0.98 ± 0.02 a	0.98 ± 0.02 a	0.98 ± 0.02 a	0.89 ± 0.04 a	0.62 ± 0.05 b
32	0.25 ± 0.04 d	0.89 ± 0.07 a	0.90 ± 0.06 a	0.90 ± 0.06 a	1.00 ± 0.00 a	1.00 ± 0.00 a	0.18 ± 0.04 c

Means in the same column followed by the same letters are not significantly different (*p* < 0.05).

**Table 3:** Linear and logistic model fitting of developmental rate against temperature for each stage of *N. penicillioides*.

Life Stage	Linear Model	R^2^	C	K	Logistic Mode	R^2^
Egg	V = 0.0084T − 0.0481	0.9611	5.73	119.05	V = 0.20/(1 + e^4.2567−0.2377T^)	0.9982
1st instar	V = 0.0319T − 0.3322	0.9790	10.41	31.35	V = 0.60/(1 + e^5.7242−0.2924T^)	0.9988
2nd instar	V = 0.0358T − 0.3225	0.9743	9.01	27.93	V = 0.73/(1 + e^4.9627−0.2574T^)	0.9989
3rd instar	V = 0.0308T − 0.3416	0.9486	11.09	32.47	V = 0.53/(1 + e^4.9613−0.2574T^)	0.9992
4th instar	V = 0.0018T − 0.2224	0.0119	-	-	V = 0.32/(1 + e^−1.4400−0.0574T^)	0.9295
pupa	V = 0.0063T − 0.0262	0.5925	-	-	V = 0.21/(1 + e^3.6178−0.2308T^)	0.9850
Egg-adult	V = 0.0021T − 0.0079	0.8752	3.76	476.19	V = 0.05/(1 + e^3.9131−0.2267T^)	0.9943

The linear model was not applicable to the 4th instar and pupa due to poor fit (R^2^ < 0.60), and the corresponding C and K values are therefore not reported.

**Table 4:** Preovipositional period, ovipostion, and longevity of *N. penicillioides* (Means ± SE).

Temp. (°C)	Preovipositional Period (Days)	Oviposition (Eggs/Female)	Longevity (Days)
17	7.90 ± 0.34 b	247.80 ± 29.10 b	112.30 ± 6.62 ab
20	6.5 ± 0.43 b	343.30 ± 35.68 a	128.40 ± 9.16 a
23	6.8 ± 0.39 b	209.00 ± 30.78 b	91.10 ± 7.01 bc
26	7.6 ± 0.40 b	252.40 ± 25.24 b	85.00 ± 8.84 d
29	12.9 ± 0.64 a	53.90 ± 15.93 c	72.00 ± 7.77 d
F; *p*	33.265; <0.001	14.12; <0.001	8.01; <0.001

Means in the same column followed by the same letters are not significantly different (*p* < 0.05).

**Table 5:** Life table parameters of *N. penicillioides* at different temperatures (Means ± SE).

Temp. (°C)	Intrinsic Rate of Increase	Net Reproductive Rate	Generation Time	Finite Rate of Increase
17	0.056	87.30	79.72	1.058
20	0.069	132.45	70.98	1.071
23	0.081	86.75	55.41	1.084
26	0.091	105.48	51.33	1.095
29	0.056	13.68	46.32	1.058

## Data Availability

The data presented in this study are available on request from the corresponding author.
